# Signatures of conserved and unique molecular features in Afrotheria

**DOI:** 10.1038/s41598-020-79559-6

**Published:** 2021-01-13

**Authors:** Arangasamy Yazhini, Narayanaswamy Srinivasan, Sankaran Sandhya

**Affiliations:** Lab 103, Molecular Biophysics Unit, Indian Institute of Science, Bangalore, Karnataka 560012 India

**Keywords:** Computational biology and bioinformatics, Evolution

## Abstract

Afrotheria is a clade of African-origin species with striking dissimilarities in appearance and habitat. In this study, we compared whole proteome sequences of six Afrotherian species to obtain a broad viewpoint of their underlying molecular make-up, to recognize potentially unique proteomic signatures. We find that 62% of the proteomes studied here, predominantly involved in metabolism, are orthologous, while the number of homologous proteins between individual species is as high as 99.5%. Further, we find that among Afrotheria, *L. africana* has several orphan proteins with 112 proteins showing < 30% sequence identity with their homologues. Rigorous sequence searches and complementary approaches were employed to annotate 156 uncharacterized protein sequences and 28 species-specific proteins. For 122 proteins we predicted potential functional roles, 43 of which we associated with protein- and nucleic-acid binding roles. Further, we analysed domain content and variations in their combinations within Afrotheria and identified 141 unique functional domain architectures, highlighting proteins with potential for specialized functions. Finally, we discuss the potential relevance of highly represented protein families such as MAGE-B2, olfactory receptor and ribosomal proteins in *L. africana* and *E. edwardii*, respectively. Taken together, our study reports the first comparative study of the Afrotherian proteomes and highlights salient molecular features.

## Introduction

The Afrotheria superorder represents a diverse group of mammals that differ distinctively from each other^1^. It contains the six orders of the elephants (Order Proboscidea), sea cows (Sirenia), hyraxes (Hyracoidea), aardvark (Tubulidentata), elephant shrews (Macroscelidea), golden moles and tenrecs (Afrosoricida). This diverse group of animals show distinct differences in sizes, preferred habitats, diets etc. Although these species bear little resemblance to each other, molecular data offers evidences for their evolutionary relationships^2^. Afrotheria is a peculiar clade characterized by lack of testicular descent through the loss of functional forms of RXFP3 and INSL3 genes^3^. Unique features such as abundant copies of tumor suppressor TP53 gene, olfactory receptors and leukemia inhibitory factor are characteristic of elephants^4–6^. Likewise, studies within Afrotheria show that the elephant and its closely related species, manatee, are associated with loss of genes involved in bile acid synthesis and low metabolic rates to maintain body temperature^7–9^. Others such as the cape golden mole are known for their high sensitivity to seismic vibrations^10^. The diverse biology of Afrotheria, coupled with the availability of substantial genomic and cognate protein sequence data now enables comparative studies of Afrotherian proteomes. Such studies are useful to understand Afrotherian genome evolution and will provide the platform to probe for potential molecular signatures that can underlie their evolutionary adaptations.

Protein-centric analysis involving Afrotherian species have mainly focused on understanding species phylogeny^1^. Detailed comparative studies involving the distribution of various functional and structural domains within the Afrotheria could help to recognize protein domains that are specifically amplified or occur in distinct combinations. Considering that Afrotheria is a unique clade of extremely divergent species with distinct phenotypes, such a comparative analysis on protein sequences could shed light on characteristic molecular features of Afrotherian species that could influence their evolutionary adaptations.

In this study, we used several comprehensive and complementary approaches to compare the proteomes of six Afrotherian species namely African elephant (*Loxodonta africana*), Florida manatee (*Trichechus manatus*), aardvark (*Orycteropus afer*), cape golden mole (*Chrysochloris asiatica*), cape elephant shrew (*Elephantulus edwardii)* and lesser hedgehog tenrec (*Echinops telfairi*). Specifically, we adopted a protein-centric approach to compare their proteomes and analysed the number and nature of orthologous proteins, extent of sequence conservation and probed for species-unique proteins. In addition, we have performed and compared domain assignments of these proteomes to determine if any specific domain expansions are observed within the Afrotherian species studied here. It is well recognized that multi-cellular metazoans and eukaryotes share similar domains but achieve functional variety through domain accretions and differences in domain architectures^11^. Therefore, we specifically probed for unique domain architectures in the Afrotherian proteomes for signatures that may potentially contribute to phenotypic variability. Our assessments show that certain domain families are highly represented in individual Afrotherian species. In addition, some domain combinations are unique to Afrotherian species and hitherto unseen in other organisms. These findings have broad implications on the evolutionary adaptations of these species that are distinct not only from each other but also from other non-Afrotherian mammals.

## Results

### Afrotherian proteome sequences are well conserved

Phylogenetic studies on the evolution of Afrotheria have shown that their origin is monophyletic^2^. In the study here, we compared proteomes of six Afrotheria to recognize similarities and dissimilarities at the protein sequence level and probed for general trends in their sequence distribution. For this, we employed 108,383 canonical Afrotherian protein sequences and performed rigorous homology searches in the non-redundant protein sequence database (NRDB) (details in “Methods” section) and screened for hits within the proteomes of the six species. We observe that for 99.5% of the query proteins, homologues were detected in one or more of the six species. Most of the proteins are well conserved, as evident from the average sequence identity of 86% among Afrotherian proteomes. However, the extent of average sequence similarity varies among individual pairs of species (Table 1). For example, based on all-protein comparisons between species, *L. africana* was found to share average sequence identity of 89% with *T. manatus*, whereas it shares 84% identity with *E. edwardii*. Of the six organisms studied here, *E. telfairi* shares the least sequence identity (83–85%) with all other Afrotherian species, affirming that it is the most distant member among the species studied here^2^. When compared with a non-Afrotherian species such as *H. sapiens*, the average sequence identity is 84% with fewer homologous proteins between them. Additionally, we observe that the average pairwise sequence identity matrix of the Afrotherian proteomes is asymmetric (Table 1). We reason that this might be due to differences in the rates of sequence divergence in each species based on their individual evolutionary pressures, or the presence of paralogues with varying sequence identities^12^.

**Table 1:** Average pairwise sequence identity of Afrotherian proteome sequences.

Average sequence identity (%) mean ± sd	*L. africana*	*T. manatus*	*O. afer*	*C. asiatica*	*E. edwardii*	*E. telfairi*
*L. africana*	*17,718*	89 ± 11	86 ± 13	85 ± 14	84 ± 14	83 ± 14
*T. manatus*	91 ± 09	*17,376*	89 ± 10	88 ± 11	86 ± 12	85 ± 12
*O. afer*	88 ± 11	89 ± 10	*17,817*	88 ± 11	86 ± 12	85 ± 12
*C. asiatica*	87 ± 12	88 ± 11	87 ± 11	*18,433*	85 ± 13	85 ± 12
*E. edwardii*	85 ± 13	86 ± 13	86 ± 13	85 ± 13	*19,110*	83 ± 14
*E. telfairi*	84 ± 12	85 ± 12	85 ± 12	85 ± 12	83 ± 13	*17,426*

Numbers along the diagonal (in italics) indicate total number of canonical proteins considered for the homology searches.

**Figure 1:**
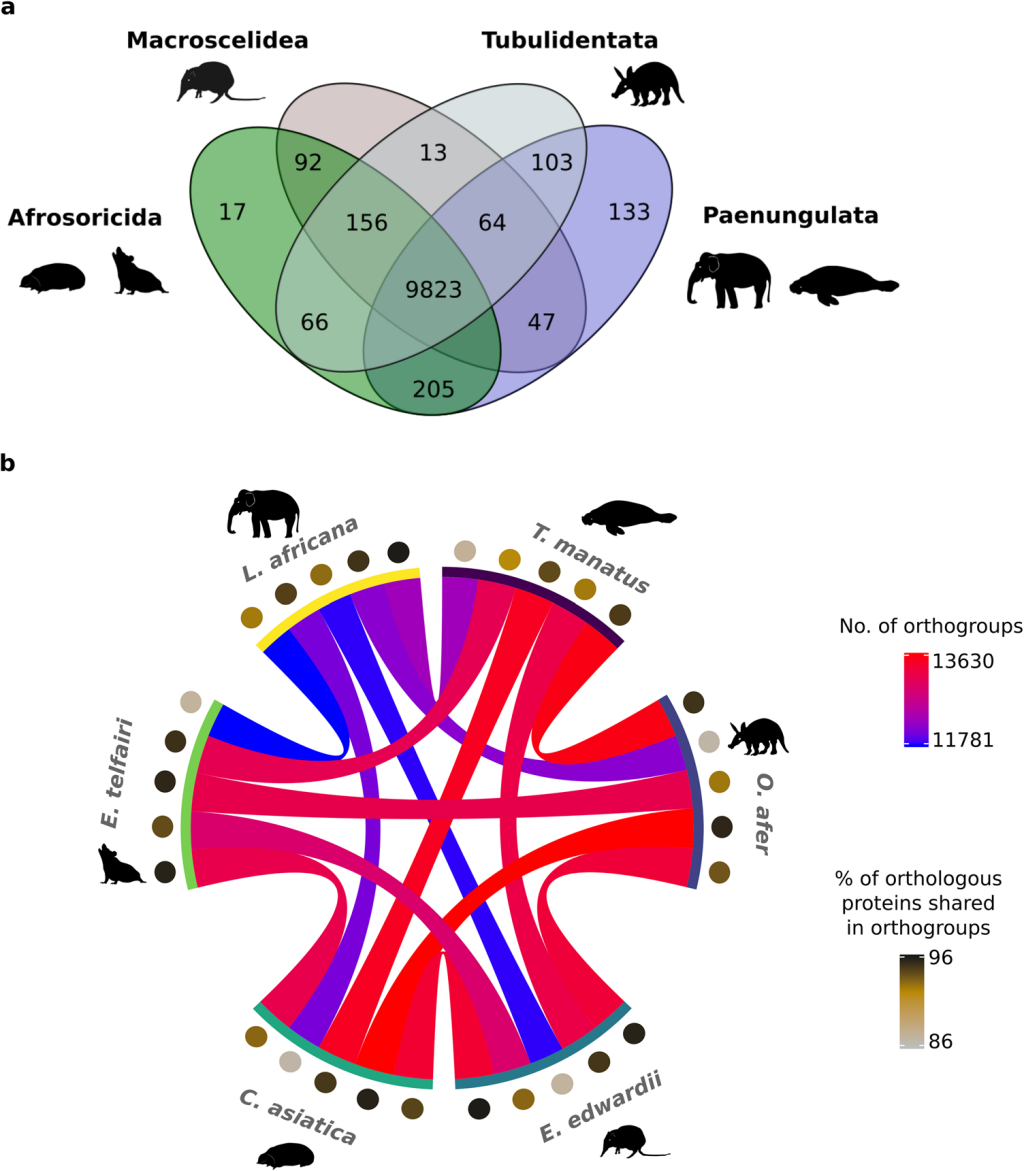
Orthologous relationships within Afrotheria. (**a**) Venn diagram shows the number of orthogroups shared among different orders of Afrotheria. Each order is represented by a different color- green (Afrosoricida), grey (Macroscelidea), blue (Tubulidentata), violet (Paenungulata). (**b**) Chord diagram^50^ that represents the total number of orthogroups shared between individual Afrotherian species. The six nodes, represented by a fragment on the outer part of the circular layout, represent each species. Color scale of the arc connecting the nodes between species (blue to red) shows the number of orthogroups between species, that may be low as in *C. asiatica* and *L. africana* or high as in *C. asiatica* and *O. afer*. Colored circles (grey-dark brown) represent the percentage of orthologous proteins present in the orthogroups between two species. Cartoon drawings of Afrotherian species were prepared using Inkscape^51^.

**Figure 2:**
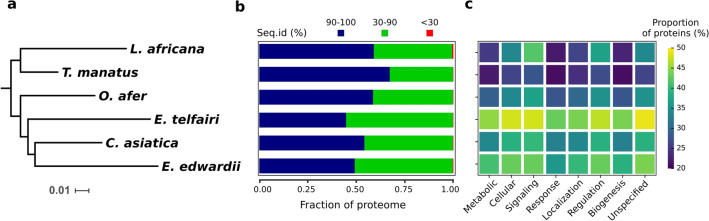
Sequence conservation in Afrotheria. (**a**) Protein sequence-based phylogenetic tree, constructed using concatenated sequence alignments of 20 different proteins, captures relationships between Afrotherian species. The bar at the bottom provides the scale of tree branch lengths. The tree was constructed using maximum likelihood (RAxML) with 1000 bootstrap replicates. (**b**) Bar plot shows the proportions of high (> 90%), moderately (30–90%) and least (< 30%) conserved proteins in the various Afrotherian proteomes (species names on the node tips of (**a**) show the Y-axis). (**c**) Heatmap shows the proportion of moderately and least conserved proteins (sequence identity range of 0–90%) from each species associated with major biological processes. Here also, species names on the node tips of (**a**) show the Y-axis. Color scale for the figure (dark blue-yellow) represents the proportion of moderately and least conserved proteins.

Within Afrotheria, it has been reported that divergence within Afrosoricida (*E. telfairi* and *C. asiatica*), Macroscelidea (*E. edwardii*), Paenungulata (*L. africana* and *T. manatus*), and Tubulidentata (*O. afer*) might have occurred very rapidly in the late Cretaceous period. Consequently, morphological evidences of their divergence might have been erased through subsequent evolution^12^. Indeed, when we examine the number of orthogroups and their orthologous proteins (details in “Methods” section), they differ within members of an order (Afrosoricida, Macroscelidea, Paenungulata and Tubulidentata). As shown in Fig. 1a, 9823 orthogroups are shared among all Afrotherian species. This number increases when we consider closer associations such as within orders. For example, within the order Afrosoricida (10,359) the number of orthogroups differs from that within Paenungulata (10,375). Interestingly, 133 orthogroups are unique to Paenungulata, while the number is ~ eightfold smaller in the Afrosoricida with only 17. Such a clustering, using orthologous proteins, is helpful to recognize proteins that are closely evolved between species. When compared across orders, these numbers capture the extent of divergence between species. For instance, higher divergence is observed between *L. africana* and *E. edwardii* or *E. telfairi* with 11,818 and 11,781 orthogroups, respectively than between *L. africana* and *T. manatus* with 12,297 orthogroups (Fig. 1b). Moreover, *L. africana* has lower number of orthogroups (11,781–12,145) shared with other order members compared to *T. manatus* (13,130–13,561), suggesting that in the Paenungulata order *L. africana* has diverged extensively (arcs are blue for *L. africana* while red for *T. manatus* in Fig. 1b). Indeed, when we consider the proportion of orthologous proteins present in the orthogroups, we find that each Afrotherian species shares lower number of orthologous proteins with *L. africana* than with other species (colored circles below species in Fig. 1b). We were able to corroborate these results on overall divergence within Afrotheria through a phylogenetic tree generated by concatenating homologues of 20 proteins (details in “Methods” section). This tree not only supports the broad grouping of the Afroinsectivora and Paenungulata, but also captures variations in sequence divergence in each species, as shown by the individual branch lengths (Fig. 2a). It is noteworthy that although every protein has its own evolutionary rate, the tree derived from concatenated and diverse protein sequences captures the overall divergence within Afrotheria supporting monophyletic origin, as also reported elsewhere using genomic sequences^2^.

Next, we focused on the number of orthologues within these six genomes (Supplementary Table S1A) (details in “Methods” section). Result shows that 61.9% of the Afrotherian proteins are orthologous and common to all six species. When we analysed their biological functions using gene ontology (GO) terms, we find that these orthologous proteins are predominantly implicated in metabolic processes and cellular regulatory mechanisms (Supplementary Table S1B). To study their extent of conservation, we binned the orthologues into three groups based on sequence identity. Majority of the orthologues in Afrotheria (52%) show very high sequence identity and are hence ‘most’ conserved (> 90% sequence identity) (Table S1B–G). 48% of these proteins are ‘moderately’ conserved (30–90% sequence identity) and primarily involved in signalling processes. Only 12 proteins share < 30% identity. Interestingly, six of these are annotated as predicted vomeronasal type 2 receptors that detect pheromones^13^ and have diverged extensively (< 30% sequence identity) within the Afrotheria (Supplementary Table S1G). Reportedly, the vomeronasal organ is absent in birds, bats, and apes but known to occur in the Afrotheria. Likely, their extensive divergence in this species reflects differences in their functional roles and sensitivity.

When we extended our analysis to include Afrotherian proteins that are homologous, we find that 47 to 69% of the six proteomes are in the ‘most’ conserved (> 90% sequence identity) category. In *E. telfairi*, 52% of the proteome shares ‘moderate’ sequence identity with their homologs. Similarly, 43 and 48% of the proteins in *C. asiatica* and *E. edwardii* share ‘moderate’ sequence identity (30–90%) with their homologs. These percentages are higher than what has been observed in *L. africana* (38%), *T. manatus* (30%) and *O. afer* (39%). Notably, we find that *L. africana* has 112 ‘least’ conserved proteins, the highest number among Afrotherian species considered here (Fig. 2b). Therefore, our homology searches reveal that majority of the orthologous proteins in these species show > 30% identity while the number of homologous proteins between these species varies. Species such as the African elephant, manatee and aardvark have a higher complement of divergent proteins when compared with the other species studied here.

To determine the functional roles of ‘moderately’ and ‘least’ conserved proteins, we associated all Afrotherian proteins with their potential biological processes using GO terms^14^. To do this, we mapped GO terms to every protein in the proteome based on gene information obtained from the respective human homolog. As a result, we could associate 169 biological processes common to about three-quarters of these proteomes. Since GO terms were available at different hierarchical levels, we traced their parent terms to the primary level for each of the biological processes and then compared the extent of protein conservation. We find that the proportion of ‘moderately’ and ‘least’ conserved proteins associated with biological processes such as signaling, response and biogenesis is higher than other primary biological processes considered here (Fig. 2c). Compared to the other Afrotherian proteomes, *E. edwardii* and *E. telfairi* both have a high proportion of ‘moderately’ and ‘least’ conserved proteins represented in all the 8 processes. On the other hand, the proportion of ‘most’ conserved proteins in almost all the biological processes is higher in *T. manatus* and *L. africana*. Here too, as in sequence divergence, the heatmap captures the extent of conservation of processes in the two broad groups of Paenungulates and Afroinsectivores. *L. africana,* has 8, 14 and 10% higher proportion of proteins in cellular processes, signaling and regulation, respectively than *T. manatus,* suggesting that *L. africana* has relatively more proteins belonging to the ‘moderately’ and ‘least’ conserved category in these processes. We also report that among the eight primary biological processes, proteins with roles in metabolism, cellular processes and response to stimulus are ‘moderately’ or ‘least’ conserved in a species-unique manner.

### Predicting the potential biological roles of Afrotheria-specific proteins

We attempted to find homologues for ~ 2% of Afrotherian proteins (2369) that are currently annotated as ‘Uncharacterized proteins’ through homology searches in NRDB and found statistically significant matches for 2185 query proteins. For 156 proteins, the homologs were detected only in Afrotherian proteomes at high sequence identity, suggesting that they are Afrotheria-specific. To improve their functional annotations, we employed an integrated approach (details in “Methods” section) and predicted function for 115 proteins (Supplementary Table S2A), many of which appear to be involved in protein (20%) and nucleic acid binding (17%). For the remaining 41 proteins, we were unable to improve existing annotation of their biological roles, suggesting that either these proteins are unique to Afrotheria or have diverged extensively when compared to their non-Afrotherian counterparts.

**Figure 3:**
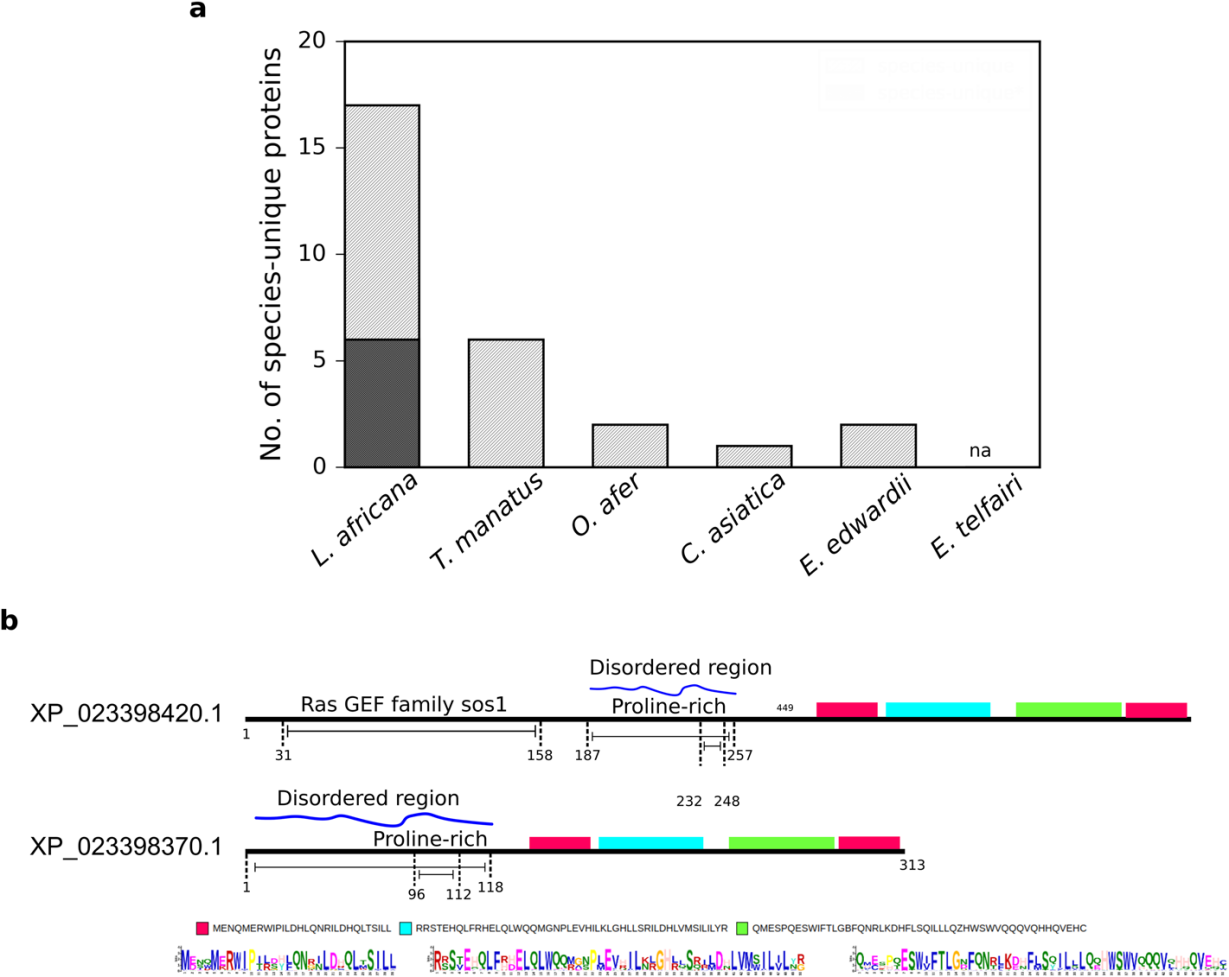
*L. africana* has the highest number of species-unique proteins in Afrotheria. (**a**) Bar plot shows the total number of species-unique proteins in each of the Afrotherian species (‘/’ symbol filled). Using a combination of approaches, function was predicted and improved for 6 of the 17 *L. africana*-unique proteins (crossline filled) (**b**) Sequence signatures of XP_023398370.1, an *L. africana*-unique protein (bottom) and its closest paralog—RGL protein (XP_023398420.1) (top) show similarities that reveal a potential role in protein-binding. Sequence motifs highlighted in different colors are common motifs shared between the paralogs that were recognized using MEME algorithm^52^. Each colored motif is expanded at the bottom of the figure.

For 28 proteins, we were unable to find any homologues even among the Afrotherian proteomes and hence refer to them as ‘species-unique proteins’ (Fig. 3a and Supplementary Table S2B) that are specific to any one of the species studied here. We probed for the presence of transmembrane regions, signal peptides, disordered regions, functional and structural domains in these sequences. Three-dimensional structure and potential molecular functions were also predicted using established methods^15,16^. To assess whether these sequences were difficult to annotate due to low complexity, we tested for this feature using a standard program and find that none were predicted to be rich in such regions. Based on a consensus of results from our integrated analysis, we have predicted secondary structure and broad molecular function for six of the 17 *L. africana*-unique proteins (Figure S1a and Supplementary Table S2). Further, we probed for paralogues for these sequences within each species, to examine if they might offer potential functional clues. For XP_023398370.1 (*L. africana)*, we identified the ral-guanine nucleotide dissociation stimulator-like (RGL) protein (XP_023398420.1) as a paralog at 97% sequence identity, covering full length of its sequence. Motif recognition in RGL and XP_023398370.1 predicted that both sequences contain disordered and proline-rich regions in addition to conserved sequence motifs (Fig. 3). We find, however, that XP_023398370.1 lacks a structural domain at the N-terminal called ‘Ras GEF family sos1’. When we examined the cognate nucleic acid sequence, we find that a retro-transposable element, homologous to the human reverse transcriptase of retrotransposon (FLJ45337), is located adjacent to the gene encoding XP_023398370.1 (Figure S1b). Since, retrotransposons mediate gene duplication, it is likely that the RGL gene might have undergone partial duplication in *L. africana*. Therefore, based on this paralogous relationship to RGL, we predict that XP_023398370.1, which is unique to *L. africana,* may have a role in protein-binding (Supplementary Table S2). Examples such as this demonstrate that gene duplication followed by sequence divergence might result in unique proteins in a species with no obvious homologue in other species.

### Functional domain distributions in Afrotheria: probing for lineage-specific domain expansions

**Figure 4:**
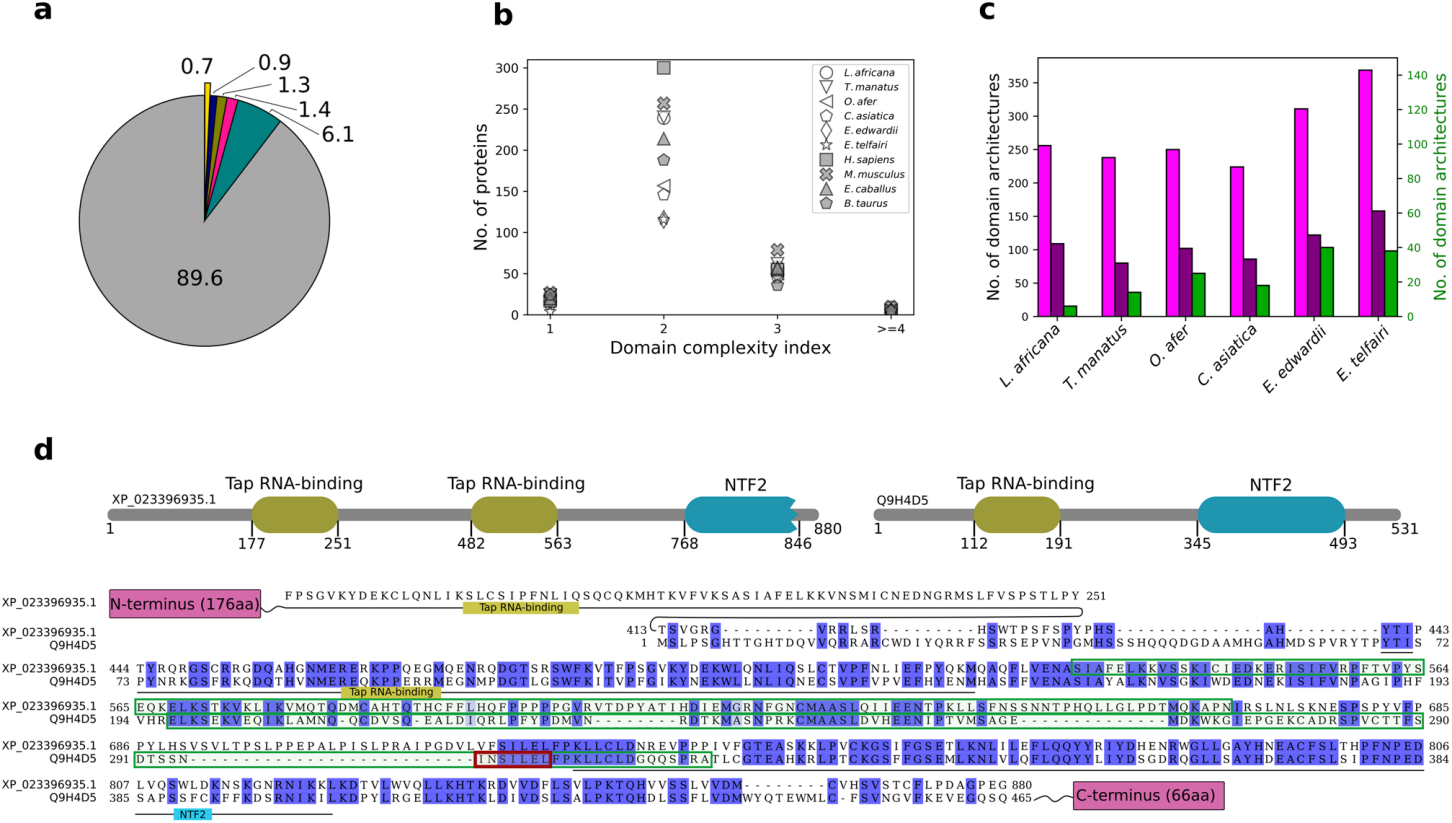
Distribution of functional domains in Afrotheria. (**a**) Pie chart shows the proportion of functional domains (in %) that are present in all (grey), in five (cyan), in four (pink), in three (olive), in two (navy) and in one (gold) of the Afrotherian species.  89.6% of all the total assigned functional domains are present in all the species while 0.7% of assigned domains are unique to any one species. (**b**) shows the distribution of KRAB domain containing proteins of varying domain complexity in various species. The number of these proteins varies most in proteins with domain complexity 2. (**c**) Bar plot shows the number of unique domain architectures in each of the Afrotherian proteomes (light magenta). Dark magenta bars show the number of unique architectures when tethered and repeat domains are not considered. Green bars, with y-axis scale on the right (in green font), show the number of novel domain architectures in each species, that are reported for the first time in Afrotheria through this study. (**d**) Domain assignments of a predicted *L. africana* nuclear RNA export 3 protein/NXF3 (XP_023396935.1) and its human homolog NXF3 (Q9H4D5). The limits of the domains are indicated by the residue positions on the assigned domains. Shown below is the sequence alignment of the two proteins that does not involve the residues 1–413 in XP_023396935.1, which harbors an additional TAP-RNA binding domain in XP_023396935.1. Identical positions in the two sequences are highlighted in blue. An exportin Crm1-binding motif present in human protein, that is also conserved in XP_023396935.1 is enclosed in a red box, conserved leucine-repeat regions in green boxes.

The availability of sensitive domain recognition methods enables the comprehensive enumeration of protein domains in each proteome and facilitates characterization of trends in their distributions. Such analysis might offer clues on the selection of domains to meet functional requirements specific to an organism. Therefore, we predicted domain make-up of each protein in our dataset to examine if specific domains are over-, under-, or unrepresented in any of the proteomes. Domain assignments were performed using hmmscan against the Pfam database. We find that out of a total of 16,712 Pfam families, 6068 are associated with nearly 92% of the proteomes showing that their coverage is uniform amongst Afrotheria (Supplementary Table S3A). More than 3300 domains are associated with each of the six proteomes (*L. africana*: 3458, *T. manatus*: 3457, *O. afer*: 3468, *C. asiatica*: 3453, *E. edwardii*: 3405 and *E. telfairi*: 3386), of which 3187 domains are common to all. The rest of the domains are common to a few or unique to a species (Fig. 4a). Further, we find that at least 33 domains are observed to occur in high numbers in one or more Afrotheria than when compared to the reference dataset (Supplementary Table S3A). These include domains such as the olfactory receptor domain that occurs in 1224 proteins of *L. africana* (only 230 in humans). Similar trends are observed in the “Nine Cysteines Domain of family 3”, GPCR, G-protein coupled receptor (*E. telfairi*-18: *H. sapiens*-7), MAGE family (Melanoma Antigen Gene) (*L. africana*-27: *H. sapiens*-13), Prothymosin (*C. asiatica*-14: *H. sapiens*-2) and other domains, suggesting unique functional roles for these domains in Afrotherian species. Additionally, “Nineteen complex-related protein 2”, Pacifastin inhibitor (LCMII), Cancer susceptibility candidate 1, 4Fe-4S single cluster domain, Nucleoporin FG repeat region and few other domains also appear to be exclusive to Afrotheria. Over-representation of a domain might point to its recruitment towards specific functional roles, although this remains to be verified.

Our assignments also highlight functional domains that occur in multiple copies in the reference proteomes but not in Afrotheria (Supplementary Table S3A). For instance, we observe that the Olduvai domain, known to show human lineage-specific expansion with roles in brain evolution, is absent in Afrotheria. GAGE domain-bearing proteins, with roles in germ cell biology, are present in higher numbers in humans than in Afrotheria. Likewise, some functional domains are completely absent in Afrotheria while present in reference species. Two of these families are DUFs (PF15488 and PF15852) that occur in Boroeutheria but not in the Afrotheria. Three other families viz. Chmadrin, “Follicular dendritic cell secreted peptide” and “Sperm acrosome-associated protein 7” are known to occur in mammals with potential roles in the organization of chromatin structure, immune response and in fertilization, respectively. These domains are absent in the Afrotherian proteomes as well. In addition, we notice that 30 functional domains are present only in some reference species. Interestingly, 16 are exclusive to humans and not seen in any of the other species. These include B melanoma antigen family, Kidney-associated antigen 1, Statherin and p53-regulated apoptosis-inducing protein 1. Our observations suggest that although the overall composition of functional domains in Afrotheria and reference proteomes are similar, few functional domains are selectively distributed among the species studied here.

### Domain architecture comparisons in Afrotheria reveal combinations unique to Afrotheria

We compared domain architecture of Afrotherian proteins to probe for novel domain combinations and species-unique trends, if any, in their distribution. For the dataset of 105,534 proteins for which functional domains were assigned, we find that 31% of the query proteins are single domain proteins (with domain assignments covering the full length of the proteins) while 69% are multi-domain proteins. However, in some instances, assignments covered only a portion of the entire length of the protein leaving the rest unassigned. We believe that such regions might harbour domains that have either diverged extensively or are yet to be identified and characterized. Hence, we considered a protein with at least 100 residues of unassigned region as a potential multi-domain protein apart from proteins where more than one domain could be identified. In doing so, we observe that the proportion of multi-domain proteins in Afrotheria increases with *E. edwardii* containing 72% multi-domain proteins, while *L. africana* has 75% (data not shown).

Protein function is guided by its constituent domains^17^. Differences in domain architectures among homologous proteins are usually functionally relevant since domain accretion can bring about lineage-specific and complex domain architectures^11^. Therefore, we assessed protein complexity based on domain composition and the linear order of domains (domain arrangement) from the N–C terminus of a protein. Here, we defined protein complexity as the total number of distinct domains present in a protein and assigned it a complexity index. Our results show that proteins with only one distinct domain are very common (~ 6642) in the dataset, suggesting that a large contribution to the multi-domain nature of the proteins is made by repetitive domains or unassigned regions. This trend holds true for reference proteomes as well. Our results show that a significant contributor to single domain proteins showing a complexity index of 1 in *L. africana* (Figure S2), is the G-protein coupled-receptor domain involved in olfaction. When we exclude this family from our calculations, we find that the number of proteins with complexity index 1 is comparable across Afrotherian species (Figure S2). Our attempts to group the proteins in this manner are useful to capture the frequencies of various domain combinations across the Afrotheria and present an opportunity to compare them with the reference proteomes. The abundance and domain combinations of the Kruppel associated box (KRAB) domain that is involved in transcriptional repression, for example, varies among the Afrotherian species. These domains are found to occur predominantly in two or three-domain containing multi-domain proteins. In *O. afer*, *C. asiatica*, *E. edwardii* and *E. telfairi* the number of proteins with this domain complexity is in the range of 110–160 which is 33% less than in *L. africana* or *T. manatus* (~ 240) and ~ 50% lower than in *H. sapiens* (Fig. 4b). Moreover, this domain is found to occur in 74 combinations in proteins with complexity index 2, 47 with complexity index 3 and 40 with complexity index 4 in the *L. africana* proteome alone (Supplementary Table S3B–D). In addition, our attempts have also served to recognize domain combinations that vary in abundance across species, as in combinations such as PUB ~ zf-RanBP ~ HOIP-UBA ~ IBR (domain complexity index of 5) or in the absence of domain combinations such as DUF4704 ~ DUF4800 ~ PH_BEACH ~ Beach ~ WD40 (domain complexity index of 5) in them (Supplementary Table S3D). These species-unique domain combinations, at varying degrees of complexity, reflect differences in domain architecture between species that may contribute to their uniqueness and represent lineage-specific domain combinations.

Secondly, we compared complexity in terms of the order in which functional domains are arranged from N- to C-terminus. For this analysis, partial domain associations were also included in defining domain architecture. In total, 10,112 domain architectures are observed, of which 1648 are unique to any one of the Afrotherian species and hence represent species-unique architectures (Fig. 4c). Noticeably, *E. edwardii* (369) and *E. telfairi* (311) possess higher number of unique architectures as compared to other species (< 260). This trend holds true with the exclusion of cases resulting from tethered domains or domain repeats (details in “Methods” section) in which uniqueness is contributed by common factors *i.e.* tethering of additional domain(s) at either end of the common domain architecture, where only tethered domain architectures are considered or variation in the number of repeating units of a domain (Fig. 4c). We note that architectures involving Immunoglobulin I-set, calcium-binding EGF domain and zinc finger C2H2 domains occur most frequently in the 405 species-unique architectures (Supplementary Table S4A). Further, when we compared these species-unique architectures with all the known architectures reported in Pfam database (version 32.0), we recognize 141 combinations that are seen in any one Afrotherian species and have thus far not been reported (Fig. 4c and Supplementary Table S4A).

The presence of many multi-domain proteins with domain complexity of 1 suggested that several repeating domains occur in the Afrotherian proteomes. Indeed, 3% of the assigned Pfam domains (i.e. 500 domains) occur as repeats with 362 domains observed commonly in all the Afrotherian proteomes. Of these, 34 domains occur as repeats in novel combinations (Supplementary Table S4B). These proteins might have unique functional roles in each species although this remains to be explored. In some cases, our efforts to characterize and compare Afrotherian proteins based on their domains was useful to predict the function of uncharacterized proteins. For example, an uncharacterized *L. africana* protein (XP_023396935.1) was predicted to possess two repeating copies of a Tap RNA-binding domain tethered with nuclear transport factor 2 (NTF2) domain. In addition, it has leucine-rich repeat regions and a protein partner interacting motif (Crm1, a nuclear export protein that aids RNA transport). Similar domain architecture is reported in Q9H4D5, a human nuclear RNA export factor 3 (NXF3) in which its Tap RNA-binding domain is known to bind snoRNAs and its NTF2 domain is known to interact with proteins to regulate trafficking of snoRNAs between nucleus and cytoplasm^18^. The extensive similarity of XP_023396935.1 with this protein suggests that it might function as a nuclear RNA export factor (Fig. 4d) as well, although the influence of additional copies of Tap RNA-binding domain on RNA export function remain unclear.

### Structural domain distributions in Afrotheria

Clearly, although the overall functional domain make-up is comparable across the Afrotheria, differences exist in their combinations that could account for proteins responsible for specific adaptations or the evolution of new functional systems. We sought to analyze the extent of structural diversity within the Afrotherian proteomes by associating domains in the ‘Structural Classification Of Proteins’ (SCOP) database to each Afrotherian protein using the SUPERFAMILY database (details in “Methods” section) and covered nearly three-quarters of the Afrotherian proteome sequences. To obtain a view of the overall distribution of the known structural classes, we determined the fraction of structural domains from each of the eight SCOP classes in the proteomes. We found that structural domains from the all-α and all-β classes were observed in 40% of the proteomes while domains from α/β and α + β classes are present in 35% of proteomes (Figure S3a). Indeed, 80% of the top 25 most abundant structural domains in the Afrotheria belong to these four major structural classes (Supplementary Table S5A). Interestingly, domains from the small-proteins class occur in 12% of the Afrotherian proteomes and yet they constitute one of the highest populations (24%) in the total structural domains associated with the proteomes (Figure S3a). This shows that although domains of small-proteins class are scarcely distributed in the proteomes, they are a large, diverse set of distinct domains. Similar assessments on the reference proteomes (accounting for ~ 73% of the proteomes of *H. sapiens*, *M. musculus*, *E. caballus* and *B. taurus*) showed that the nature and distributions of structural domains are similar between Afrotheria and reference proteomes.

**Figure 5:**
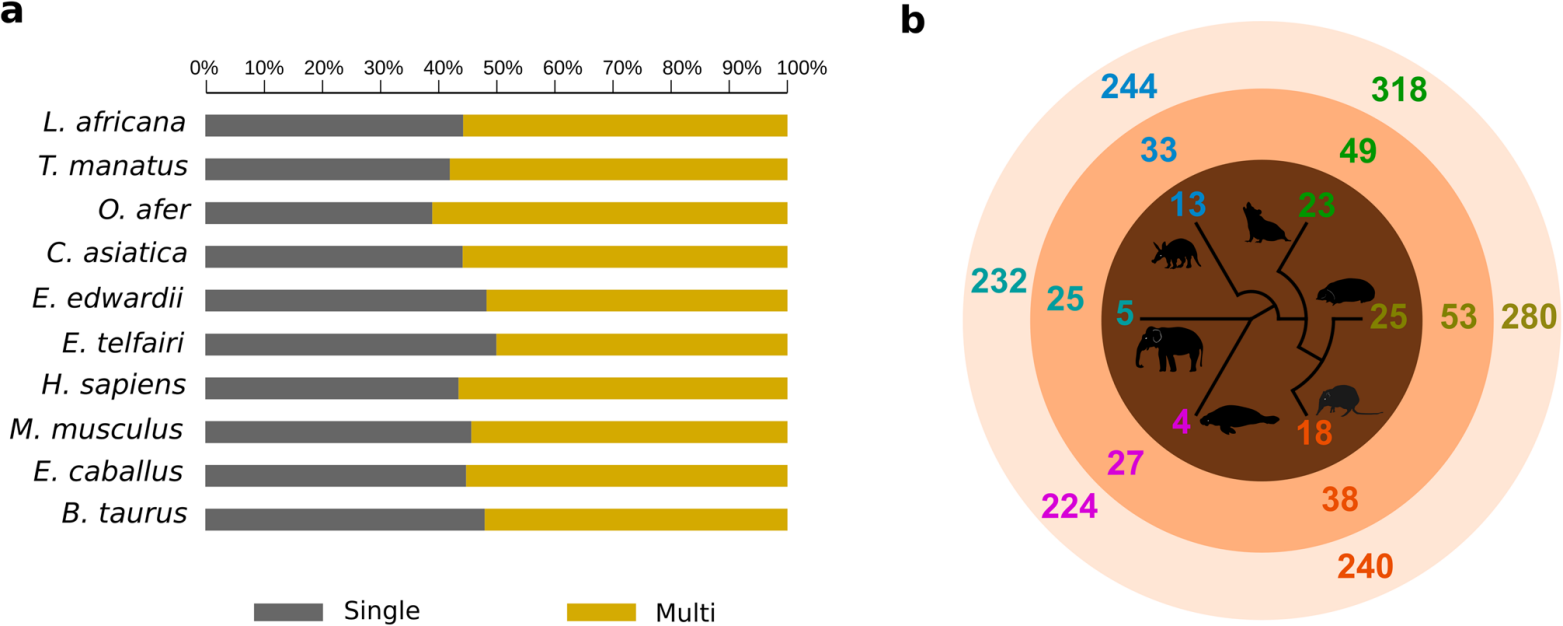
Distribution of structural domains in Afrotheria. (**a**) Bar plot shows the proportion of SCOP ‘multi-domain’ class in single (grey) and multi-domain (gold) proteins in various species. (**b**) Distribution of structural domain architectures in Afrotherian proteomes. Numbers in the outer circle (light pink) represent the total number of unique domain architectures observed in *L. africana* (blue), *T. manatus* (pink), *O. afer* (dark blue), *C. asiatica* (orange), *E. edwardii* (gold) and *E. telfairi* (green) upon comparisons within Afrotheria. Numbers in the middle circle (orange) show the number of unique domain architectures in each Afrotherian species, when architectures resulting from tethered domains and domain repeats are excluded from the analysis. The innermost circle (brown) shows the number of novel structural fold combinations in each species. These numbers are projected on a phylogenetic tree of the 6 species, derived using a concatenated sequence alignment of 20 proteins as in Fig. 2a. Cartoon drawings of Afrotherian species were prepared using Inkscape^51^.

We also investigated the relationship between domains of each structural class and their occurrence in single and multi-domain proteins. Results show that except membrane-domain containing proteins, which are typically transmembrane receptors, domains belonging to all other structural classes are predominantly present in proteins having multiple domains (Figure S3b). The proportion of the SCOP class ‘multi-domain’ varies in the Afrotheria with highest in *E. edwardii* (230) and least in *L. africana* (203). Such ‘multi-domain’ class proteins are predominantly associated with GO terms that are involved in processes such as negative regulation of fibrinolysis, blood coagulation, fat cell proliferation and platelet activation. Overall, when we consider the proportion of the multi-domain proteins having this class, we find it highest in *O. afer* (61%) and least in *E. telfairi* (50%), (Fig. 5a).

At the superfamily level, the SCOP database groups together protein domain families with broadly conserved function and potentially common evolutionary origin. Of the 1170 domain superfamilies that we assigned; 1067 domains are common to the six Afrotherian species. We analysed their structural domain combinations and identified 5494 architectures of which only 682 are present in all the Afrotheria. For example, Concanavalin A-like lectins/glucanases domain, primarily involved in carbohydrate binding, are seen in all Afrotheria, and observed in 87 species-unique domain architectures (Supplementary Table S5B). As in the results from our studies on functional domains and their combinations, *E. telfairi* shows the highest number (318) of species-unique structural domain architectures (Fig. 5b). We find from the domain assignments, that differences in the number of copies of the domains assigned to a protein and the presence of such domains in the context of tethered domains account for these high numbers. When we exclude such entries, we find 225 unique domain architectures in the Afrotheria (Fig. 5b). Of these, 115 are domain combinations that have not been observed before in any species and are reported here for the first time (Supplementary Table S5C). When we consider these associations in terms of their structural folds, we find 88 novel fold combinations in Afrotheria (Fig. 5b). Further, 36 of them are supported by functional information based on the integrated annotation approaches applied here (Supplementary Table S5C). Predictions of biological role, based on GO ontology terms, suggest that they are involved in regulatory mechanisms and intracellular processes such as signal transduction, nucleic acid binding, protein modifications, cell cycle and transport.

### Domains that occur in high numbers in Afrotheria

**Figure 6:**
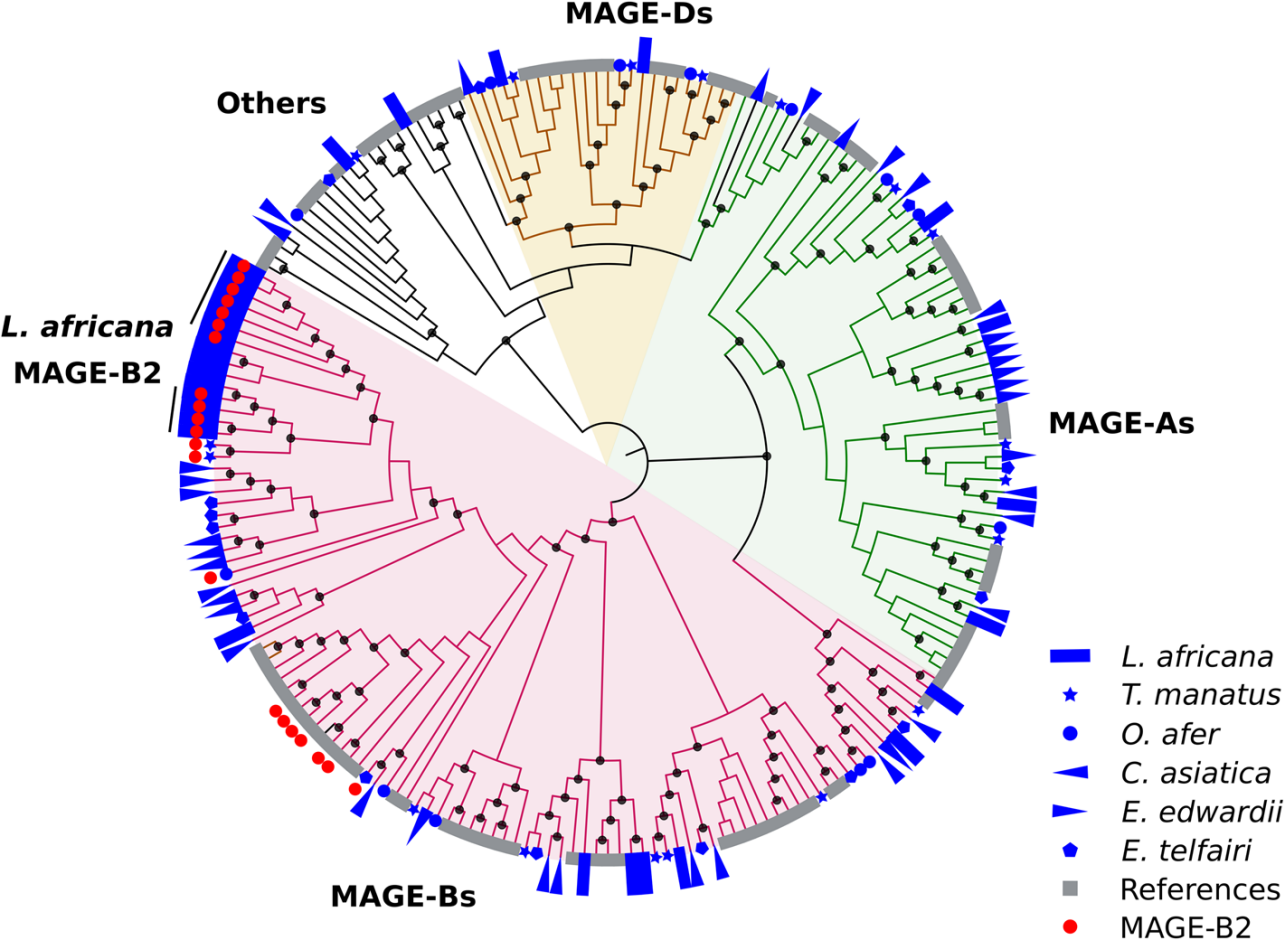
Unusual representation of MAGE family proteins in Afrotheria. Phylogenetic tree (generated by Neighbor-Joining method (using 332 aligned positions with 2000 bootstraps^53,54^) depicts the number and relationships of MAGE domain family containing proteins present in Afrotheria (filled blue symbols) and reference proteomes (filled grey squares). Each Afrotherian species is depicted with a different symbol- *L. africana* (rectangle), *T. manatus* (star), *O. afer* (circle), *C. asiatica* (inward triangle), *E. edwardii* (outward triangle) and *E. telfairi* (pentagon). Tree branches are colored as green, pink, and brown with similar color background, to show the distribution of MAGE proteins corresponding to subfamilies A, B and D, respectively. Branches colored black correspond to proteins belonging to other categories of MAGE family (E, G, Nectin etc.). MAGE-B2 proteins, highlighted with filled red circles, show a higher number in *L. africana* than in all other species considered here.

Some domain families are found in large numbers in Afrotherian proteomes suggesting that proteins harbouring such domains might have been selectively recruited for various functions. Comparison of functional and structural domain distributions among Afrotheria revealed that melanoma-associated antigen (MAGE) homology domain is one such domain, found to occur in 30 proteins in *L. africana*. This number is higher than in *T. manatus*, *O. afer*, *C. asiatica*, *E. edwardii* and *E. telfairi* that have fewer proteins with this domain (18, 13, 19, 17 and 14 proteins, respectively). MAGE proteins are a highly conserved group of proteins in eukaryotes that are primarily known as cancer antigens, due to their abundance in various cancers^19^. They are grouped into ten subfamilies under two types (I and II) depending on where they are expressed. Type I MAGE (includes subfamilies A, B and C) are expressed only in testis and their somatic cell expression is associated with cancers. Type II MAGE proteins (D-L and Nectin) are implicated in the regulation of neuron differentiation and apoptosis^19^. We observed that MAGE-B (type I) family proteins are highly represented in Afrotheria and reference species (Fig. 6 and Supplementary Table S6A). In particular, MAGE-B2 is the most abundant subfamily in *L. africana* (11 proteins) (filled red circles in Fig. 6). In contrast, only two or a single copy of MAGE-B2 proteins is observed in *T. manatus, O. afer* and *E. telfairi* while not detected in other Afrotherian species. Among the reference proteomes, *B. taurus* possesses the highest number of MAGE proteins although it has only six MAGE-B2 proteins^19^. In *L. africana*, genes for these proteins are located consecutively within two scaffolds in the genome assembly (NW_003573459.1 and NW_003573502.1), suggesting that MAGE-B2 family genes might have extensively duplicated. The physiological function of MAGE-B2 family proteins expressed in spermatids during post-meiotic stages of spermatogenesis, is to protect the germline under nutrient stress conditions^20^. It promotes cell proliferation upon binding with histone acetylase 1 that activates E2F transcription activation. *L. africana* that is slow to breed likely needs a constant production of high-quality gametes. Therefore, one plausible role for this protein, which is restrictively expressed in testis, might be to assist in cell proliferation and gamete protection.

**Figure 7:**
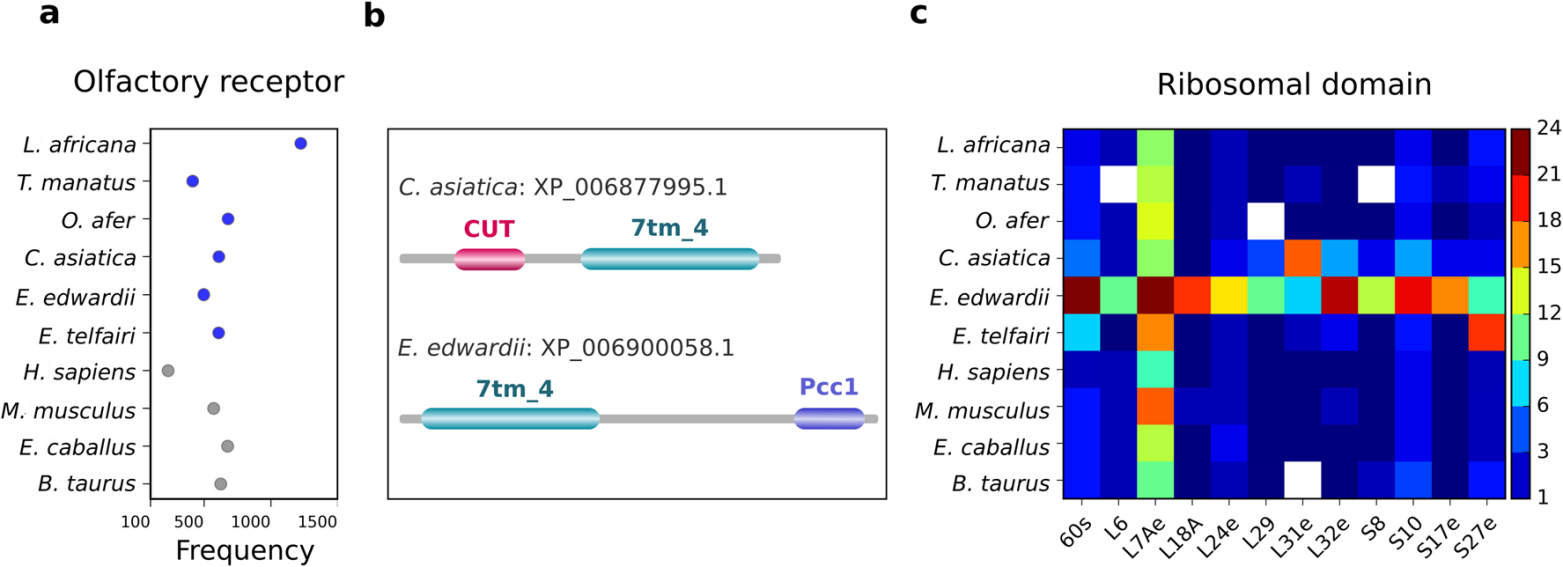
Domains that are unusually represented in Afrotherian proteomes. (**a**) Dot plot showing the frequency of olfactory receptor domain containing proteins in various Afrotherian species (in blue) and reference species (in grey). Frequency is normalized by the total number of proteins with functional domain assignments. (**b**) Novel domain architectures of olfactory receptor domain (7tm_4) with CUT domain observed specifically in *C. asiatica* (XP_006877995.1) and Pcc1 domain in *E. edwardii* (XP_006900058.1). (**c**) Heatmap shows the abundance of ribosomal domain containing proteins in various species. Color scale (blue to maroon) shows the variation in number of each protein. *E. edwardii* shows high numbers for all 12 proteins listed here. White bars indicate absence of a particular protein in the species.

Seven transmembrane G-protein coupled receptor family proteins (G-protein coupled receptors) are involved in sensory mechanisms and their gene copies are known to be extensively expanded in some mammals^5^. Since these are of seven sub-types, we sought to determine their distribution in the Afrotherian proteomes. Here, we find that the olfactory receptor (OR) is remarkably abundant in *L. africana* as compared to other Afrotheria and the reference proteomes (Fig. 7a). Interestingly, serpentine receptors, a class of 7TM GPCR, are observed only in the *E. edwardii* (XP_006892129.1) among the Afrotheria. This protein family is known to be the major sensory system for soil nematodes, which are blind and deaf^21^, and plays an important role in chemoreception. It mediates physiological responses to diverse stimuli like light, odorants, bitter and sweet tastants, pheromones, lipids etc.^22^. Its presence in *E. edwardii,* that is predominantly active in the day and shows several adaptations related to burrowing, suggests that it may be employed to perceive chemical stimuli. Additionally, the absence of fruit fly and insect-specific odorant and chemosensory receptors in Afrotheria suggests that they are probably non-essential for the Afrotherian sensory system. Further, we find that the olfactory receptor is associated with unusual domain architectures in Afrotheria that are thus far not reported. In *C. asiatica* and *E. edwardii*, the olfactory receptor domain is associated with a DNA-binding motif called the CUT domain (XP_006877995.1) and a transcription factor Pcc1 domain (XP_006900058.1), which regulates genes involved in cell cycle progression and polarization, respectively (Fig. 7b)^23,24^. Likely, these proteins are involved in functions other than odorant binding.

114 functional domain families are reported to be associated with ribosomal proteins (Pfam 31.0 release), of which 104 are observed in Afrotherian proteomes. For twelve domains, we find that the distribution varies within Afrotheria (Fig. 7c). These include 60s, L7Ae, L18A, L24e, L31e, L32e, S17e and S27e domains that are known to be specific to eukaryotes and archaebacteria^25^. In *E. edwardii*, we find that the number of copies of these twelve domains is higher than in other Afrotheria and reference species (Supplementary Table S6B). For example, the ribosomal domain L6 occurs in 11 proteins of *E. edwardii* while in other Afrotheria and reference species, it occurs only in one protein. A few ribosomal domain containing proteins are involved in the regulation of protein translation under stress conditions^26^. Given that *E. edwardii* undergoes stress during torpor and hibernation, a plausible role for these proteins could be in translational regulation. In order to verify their relevance to stress, we surveyed the distribution of ribosomal domains in twelve animals that are known to undergo torpor/hibernation^27^ (Supplementary Table S6B). Surprisingly, we observed that few ribosomal domains (e.g. 60s, S4e and S27e) are abundant in Brandt’s bat, grey mouse lemur and few other animals suggesting a role in stress regulation.

## Discussion

Mapping morphological differences between species to variations in their genome is a daunting and challenging task. Most traits are the cumulative result of accumulation of changes in sequence or variations in the way protein domains are arranged or interact with each other^17^. Comparative genomics is an effective tool to study evolutionary history and utilizes the information on similar and dissimilar molecular signatures to identify adaptive mechanisms of species^28^. Here, we have attempted to understand the differences between Afrotheria, comprising of African-origin animals that share limited resemblance superficially. Indeed, earlier studies have reported differences in aspects such as cancer resistance, olfaction, respiration and metabolic rates etc. in these animals^5–8^. The availability of genomic data provides an opportunity to perform a comparative study based on their proteome sequences and constituent domains.

Our studies show that the overall genome size is comparable in the six species studied here, with 62% of the proteomes being orthologous. These proteins predominantly participate in well conserved processes such as metabolism and cellular regulatory processes. Among the six species, ~ 99.5% of some proteomes are homologous, implying close relationships between species. This is also observed when we group the sequences based on their sequence similarity. Indeed, our results show that *C. asiatica, E. edwardii* and *E. telfairi* have higher proportion of moderately conserved proteomes than when compared with *L. africana, T. manatus* or *O. afer*. In terms of functions, proteins that are involved in specific processes such as signaling, metabolic and cellular processes and response to stimulus have diverged extensively between *L. africana* and *E. telfairi*.

When we consider orthologues, their number and extent of sequence conservation is high between *L. africana* and *T. manatus*, both paenungulates (Fig. 1). This trend is lower when we compare the protein sequences of Paenungulates with any of the Afroinsectivores. Of the six Afrotheria studied here, *L. africana* has the most divergent and unique proteins implying that a higher proportion of the *L. africana* proteome has evolved divergently from other Afrotherian species. Indeed, the distance-based trees that we obtain based on the homology searches performed here mirrors the phylogenetic trees that have broadly classified the various Afrotheria^12^.

Our searches helped to group proteins that were uniformly unannotated with very little functional information available in the six species. For such cases, we attempted to integrate results from various approaches to infer potential biological role of Afrotheria-specific uncharacterized proteins. In *C. asiatica* and *E. edwardii* that possess many uncharacterized proteins, we were able to use orthology to predict potential function of such proteins in protein-binding and nucleic acid-binding and believe that proteome annotation for these species needs revision. One of our main goals was to identify proteins in each species with no homologs in other genomes. Such proteins might either be specific to any one Afrotherian species or common to all Afrotheria but absent in other organisms. We have provided this comprehensive list of species-specific, Afrotheria-specific proteins in this study and anticipate that it will be a valuable resource for future research of these related organisms.

One of the approaches to study genome evolution is analysis of the modular nature of proteins. Since most proteins are composed of one or many protein domains, which are the units of protein structure, function, and evolution^17,29^, we analysed the functional and structural domain make-up of the Afrotherian proteomes. The main idea here was to evaluate whether any domain is specifically expressed in high copies in the proteomes and to compare their domain architectures. Here, we find that the GPCRs are substantially well represented in the Afrotheria in general, and especially in *L. africana*. A majority of these GPCRs occur in single-domain proteins and they constitute 36% of the single domains in our dataset. We also observe that the proportion of the domains known as ‘small-proteins’ (12%) is high in Afrotheria. Previous studies have also suggested that such proteins are relatively recently evolved and might have been recruited for specialized functions^30^. The comparative studies performed here have served to enumerate not only a comprehensive list of functional and structural domains in the Afrotherian proteomes but also highlight many domains that occur in high copies in Afrotheria but not in the reference genomes or vice versa. Indeed, our detailed tables are useful to recognize examples such as the novel domain associations of the KRAB domain in Afrotheria and the relatively poorer representation of receptors such as 7tm_3, Takusan domains etc., that are highly represented in other organisms *(M. musculus)*. We provide these detailed lists of domain distributions and anticipate that they will be useful to probe for species-unique domains and further study by others (Supplementary Tables S3, S4 and S5).

It is important to note that even though 92% of the Afrotherian proteomes could be associated with functional domains, 63% of the proteins had functional domains spanning only a region of the sequence. Nearly 8% of the proteomes were not associated with any of the known functional domains suggesting that such proteins have either diverged extensively from other proteins or harbour hitherto unknown functional domains. Among the Afrotheria, we find that *L. africana* has the highest proportion of multi-domain proteins, while *E. edwardii* has the least. Further, it is well appreciated that domain rearrangements across phyla can contribute to the formation of unique domain combinations resulting in protein diversity^31^. While earlier attempts have been made to enumerate and compare the domain architectures of four Afrotherian species^32^ and recognized 181 novel combinations, our assessments are more rigorous since we considered both domain order from N–C terminus and contributions of potential domains from unassigned regions > 100 residues. We find that 1648 of the total 10,112 domain architectures observed in Afrotheria are species-unique raising the possibility that new protein functionalities might have evolved through domain combinations^17^ in these species. This, however, remains to be verified through experiments. Results obtained from our study, therefore, add new facets to novel domain combinations in many proteins and offer clues to the evolution of proteins in these six related species. Indeed, we recognize 141 domain architectures that are seen in any one Afrotherian species that have thus far not been reported in the Pfam database (version 32.0) (Fig. 4c). The presence of highly mobile domains such as immunoglobulin I-set, calcium-binding EGF domain and zinc finger C2H2^31^ in species-unique architectures highlights that apart from being abundant, their association with other domains can contribute to the domain complexity of the proteome. Additionally, the occurrence of repetitive copies of a domain might add to proteome complexity, as observed for 3% of total domains associated with the Afrotherian proteomes. Of the 225 structural domain architectures observed to uniquely occur in any one Afrotherian species (Fig. 5b), 115 domain combinations have been reported here for the first time and have not been observed before in any other species (Supplementary Table S5).

Earlier studies have reported the unusually high occurrence of the TP53 gene in the elephant genome. It is likely that they protect the elephant from cancers by increasing gene dosage or conferring adaptive benefits due to increased regulatory flexibility^6^. Recent studies have associated type I group of MAGE family proteins with spermatogenesis and protecting the germline under nutrient stress conditions^20^. MAGE-B2 proteins are abundant in *L. africana* and restrictively expressed in spermatids during post-meiotic stages of spermatogenesis^19^. We reckon that more copies of proteins with this domain may positively influence cell proliferation and gamete protection, given that *L. africana* shows low reproductive rates. *E. edwardii* is a heterothermic animal that can undergo daily torpor or hibernation^27^. Under such stress conditions, energy consuming processes such as protein translation are suppressed and certain ribosomal proteins act as translational regulators^26^. For example, ribosomal protein L13a silences translation of specific mRNAs under inflammation. Ribosomal proteins L6 and L11 interact with HDM2 protein and regulate the level of P53 under ribosomal stress condition. In our study, a few ribosomal proteins are unusually highly represented, including L6, in *E. edwardii* and *E. telfairi* (Fig. 7c). Similar copy numbers for ribosomal proteins in other species that undergo daily torpor/hibernation indicates their important roles in translational regulation under stress (Supplementary Table S6B). In summary, given our limited knowledge in the above areas, we cannot conclude that the increased number of domains may directly contribute to genotype–phenotype variations in each species but the clear differences in select domain families in Afrotheria appear to correlate well with their overall large differences.

Our studies have broad implications in furthering the understanding of a wide range of genetic and evolutionary processes among Afrotheria and other mammals, some of which have been considered as reference proteomes in this study. We anticipate that our data can serve as an important resource that is useful to researchers to perform a wide range of analysis.

## Methods

### Curation of whole proteome sequences of six Afrotherian species

Whole proteome sequences were retrieved from the NCBI database (ftp://ftp.ncbi.nlm.nih.gov/genomes/refseq/vertebrate_mammalian/) for *Loxodonta africana* (GCF_000001905.1), *Trichechus manatus* (GCF_000243295.1), *Orycteropus afer* (GCF_000298275.1), *Chrysochloris asiatica* (GCF_000296735.1), *Elephantulus edwardii* (GCF_000299155.1) and *Echinops telfairi* (GCF_000313985.1). For comparative studies, we considered only the longest translated protein sequence per gene, to minimize the uncertainties associated with expression and function of the predicted isoforms. A total of 21,094, 19,108, 19,511, 19,638, 20,443 and 18,858 curated protein sequences from *L. africana*, *T. manatus*, *O. afer*, *C. asiatica*, *E. edwardii* and *E. telfairi* were used for all analyses in this study, unless stated otherwise.

### Identification of homologs

Homology searches were performed with every Afrotherian protein sequence as a query in the non-redundant database of protein sequences (NCBI). To find orthologues, both OrthoFinder (with default parameters) and reciprocal BLASTP searches were performed among all six Afrotheria^33,34^. Low-quality and partial sequences, although a part of the database, were not employed as queries to avoid unreliable observations. Hits from BLASTP search were parsed by E-value (10^–4^) and query coverage threshold (75%). Protein queries with no homologs from Afrotheria were subjected to TBLASTN searches against genomic and mitochondrial DNA sequences of Afrotherian genomes. In such searches, queries that detected hits at E-value better than 10^–10^ and 75% query length coverage were considered as homologs.

Queries with no orthologs or homologs identified within Afrotheria were employed in searches for homologues in other eukaryotic genomes using PSI-BLAST searches against the RefSeq database (NCBI). Hits were identified at the end of four iterations and selected based on E-value (10^–4^) and query coverage threshold (75%)^34^. Additionally, for queries that did not find homologues, we performed PSI-BLAST searches without filters to account for low-complexity and compositionally biased regions. When these searches also failed, we performed HHblits against the uniclust30 (2017) database^35^ at an E-value cut-off of 10^–3^ and query coverage threshold of ≥ 60%.

We grouped the results into four categories (1) ‘Orthologs’: proteins with bi-directional hits within Afrotheria or as identified by OrthoFinder. (2) ‘Homologs within Afrotheria’: proteins with uni-directional hits (includes results from PSI-BLAST, HHblits and TBLASTN searches). (3) ‘Homologs outside Afrotheria’: proteins with hits only from non-Afrotherian species. (4) ‘Species-unique’: proteins that failed to recognize homologs in any search method.

### Phylogenetic tree construction

Protein sequence-based phylogenetic tree was constructed using concatenated sequence alignments of 20 different proteins (corresponding to genes COA7, HOXA1, HOXB1, HOXD3, TACO1, MRPL2, MRPL3, MRPL12, MRPL15, MRPL13, MRPL15, MRPL17, MRPL18, MRPL19, MRPL20, MRPL21, MRPL22, MRPS17, RPS3 and RPS6). This was performed to capture the different evolutionary rates of individual proteins^36^. Afrotherian orthologs of each protein were aligned using Clustal Omega, ignoring columns with more than 95% gaps and then concatenated with a total of 4757 aligned residue positions^37^. The tree was constructed using maximum likelihood method (RAxML program) using 1000 bootstrap replications. The best bi-partitioned tree was used to infer phylogenetic relationship^38^.

### Using gene ontology to infer biological processes

For function annotation, gene ontology terms were mapped to all six Afrotherian proteomes using the gene symbols of human homologs. Mapping was done by gProfiler using SCS algorithm for computing p-value from multiple testing correction^14,39^. Associations of gene ontology terms to proteins with p-value > 0.05 or inferences through electronic annotation (IEA) were ignored. We considered only those biological processes in which at least 75% of proteins in the process are associated with Afrotherian proteomes through GO mapping. Through direct relationships, lower level or intermediate biological process terms were mapped to their parent terms at the first level in biological process hierarchy, to compare across species.

### Functional characterization of Afrotheria-specific proteins

To improve annotation of uncharacterized Afrotherian proteins, we probed for functional details of non-Afrotherian homologs recognized through BLASTP searches. In addition, InterProScan, functional and structural domain assignments were performed for each query sequence^16,25,40,41^. Proteins with no homologs (orphan proteins) in such searches were deemed as species-unique proteins. They were screened for low complexity regions using the NCBI-seg program^42^. Transmembrane regions and signal peptides were predicted using TMHMM, TOPCON, Phobius, PrediSi, SignalP and SecretomeP programs^16,43–46^. Disordered regions, three-dimensional structure and gene ontology terms were predicted using IUPred2A and PSIPRED server^15,47^.

### Functional and structural domain assignments of Afrotherian proteomes

Functional domain assignments were performed on 108,383 Afrotherian protein sequences and 84,376 canonical proteins from *H. sapiens*, *M. musculus*, *E. caballus* and *B. taurus* that constitute our reference dataset, using hmmscan^40^ against PFAM database (version 31.0). Only reliable domain assignments were considered using default E-value and domain hmm coverage filter (60%)^25^. Common problems in assignment such as domain overlap, splitting and incomplete assignments were treated as follows (1) in case of domain overlap (overlap region > 40% of either domain), only the longer domain was considered. (2) In case of splitting with no intervening domain(s), individual domain parts were stitched together if their domain coverages were > 60%. (3) Where intervening domains were present, each of the assigned domains was considered. Partially assigned domains were considered only for studying domain architectures. Further, to quantify domain composition of individual proteins, we defined the term domain complexity index of a protein as the total number of distinct functional domains present in it.

Structural domain assignments were made by searches against the HMM models in the SUPERFAMILY database that uses SCOP domain definition^41^. Default E-value thresholds were employed to assign domains. In case of domain overlap, longer domain or domain with lower E-value was considered. In both functional and structural domain analyses, to identify multi-domain proteins, we considered 100 or more residues of unassigned region as potentially a domain. In addition, we have considered two scenarios while identifying species-unique domain architectures (i) domain tethering and (ii) domain repeat. In tethering (i), domain architecture of two proteins from different organisms can differ by the presence of another domain at either end of the common architecture (e.g. A-B vs A-B-C). Such cases may arise because of domain recombination or domain fusion. In such instances, to determine unique architecture only domain instances of A-B-C were considered in the counts, since the combination of A-B is also observed in A-B-C. In the case of domain repeat (ii), differences in the domain architectures between two proteins may result from variation in the number of copies of a domain (e.g. A-B-C vs A-B-B-C). Occurrence of the same basic unit (A-B-C) in different copies because of repeating copies of a domain were considered as domain repeats and therefore, not considered further to identify species-unique architecture.

### Prediction of functions of proteins showing unique domain architectures

Structural domains that contribute to species-unique domain architectures were associated with GO terms using domain-centric gene ontology method^48^ and superfamily annotations by Vogel and Chothia^49^. These terms were used to interpret potential functional role of proteins having unusual and novel structural domain architectures.

## Supplementary Information

Supplementary Information 1.

Supplementary Information 2.

Supplementary Information 3.

Supplementary Information 4.

Supplementary Information 5.

Supplementary Information 6.

Supplementary Information 7.

## Data Availability

All data pertaining to the manuscript have been provided in the form of figures. Supporting information has been made available as Tables S1–S6 and supporting Figures S1–S3. Datasets pertaining to the sequence searches described here, are available from the corresponding author on reasonable request.
