# Supplementary material for: Signatures of conserved and unique molecular features in Afrotheria
Source: Sci Rep. 2021 Jan 13;11:1011. doi: 10.1038/s41598-020-79559-6 (PMC7806701; doi:10.1038/s41598-020-79559-6)
Supplement: Supplementary file 1 — Supplementary Information 1. [file 41598_2020_79559_MOESM1_ESM.pdf]

Supplementary data

Signatures of conserved and unique molecular features in Afrotheria

Arangasamy Yazhini[1], Narayanaswamy Srinivasan[1*] and Sankaran Sandhya[1*]

Author Affiliations

[1]Molecular Biophysics Unit; Indian Institute of Science; Bangalore, Karnataka, 560012; India.

*To whom correspondence should be addressed.

Sankaran Sandhya, Lab 103, Molecular Biophysics Unit, Indian Institute of Science, Bangalore – 560012, Karnataka, India. Tel: +918022932837; Fax: +918023600535. Email: sandhyas@iisc.ac.in

Narayanaswamy Srinivasan, Lab 103, Molecular Biophysics Unit, Indian Institute of Science, Bangalore – 560012, Karnataka, India. Tel: +918022932837; Fax: +918023600535. Email: ns@iisc.ac.in

Supplementary figure S1. Structural annotations of six *L.africana*-unique proteins. (a) Predicted three-dimensional structure of each protein is shown using vertical bars (helices (blue), strands (red)). Protein-binding predicted disordered regions are shown using cyan bars. (b) Gene structure of *L.africana*-unique protein (bottom) and its closest homolog (top). Retro-transposable element of human (FLJ45337) sequence is homologous to the region located adjacent to the gene encoding *L.africana*-unique protein.

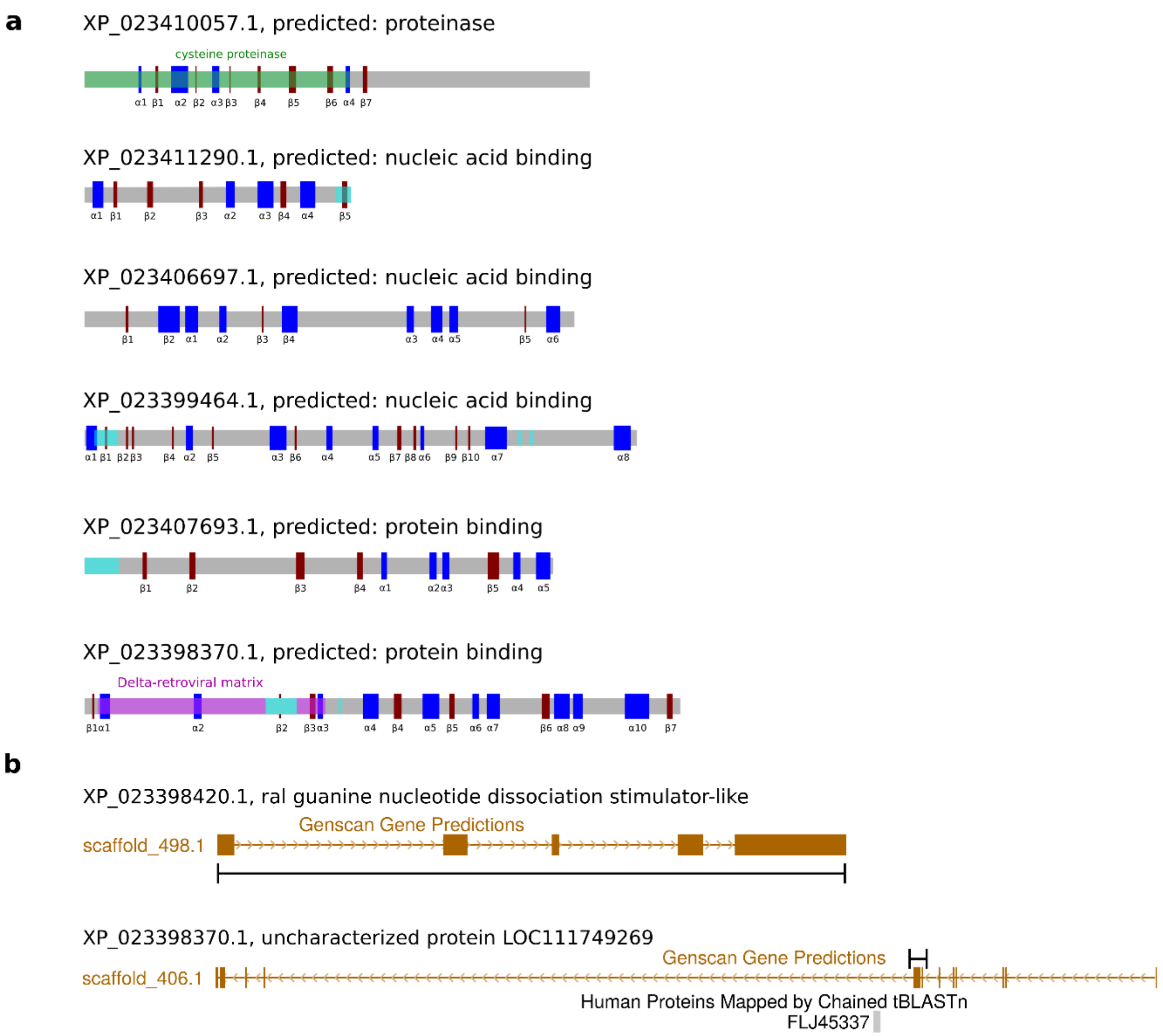

Supplementary figure S2. Functional domain complexity index of Afrotherian proteomes. Frequency distribution of functional domain complexity index, which is defined as the number of distinct functional domains present in a protein (frequency is normalized by the total number of proteins in the proteome that are associated with functional domains). Data corresponding to 'References' are averaged values from four species (*H.sapiens*, *M.musculus*, *E.caballus* and *B.taurus*). Shown at the top right corner is frequency distribution after excluding olfactory receptors.

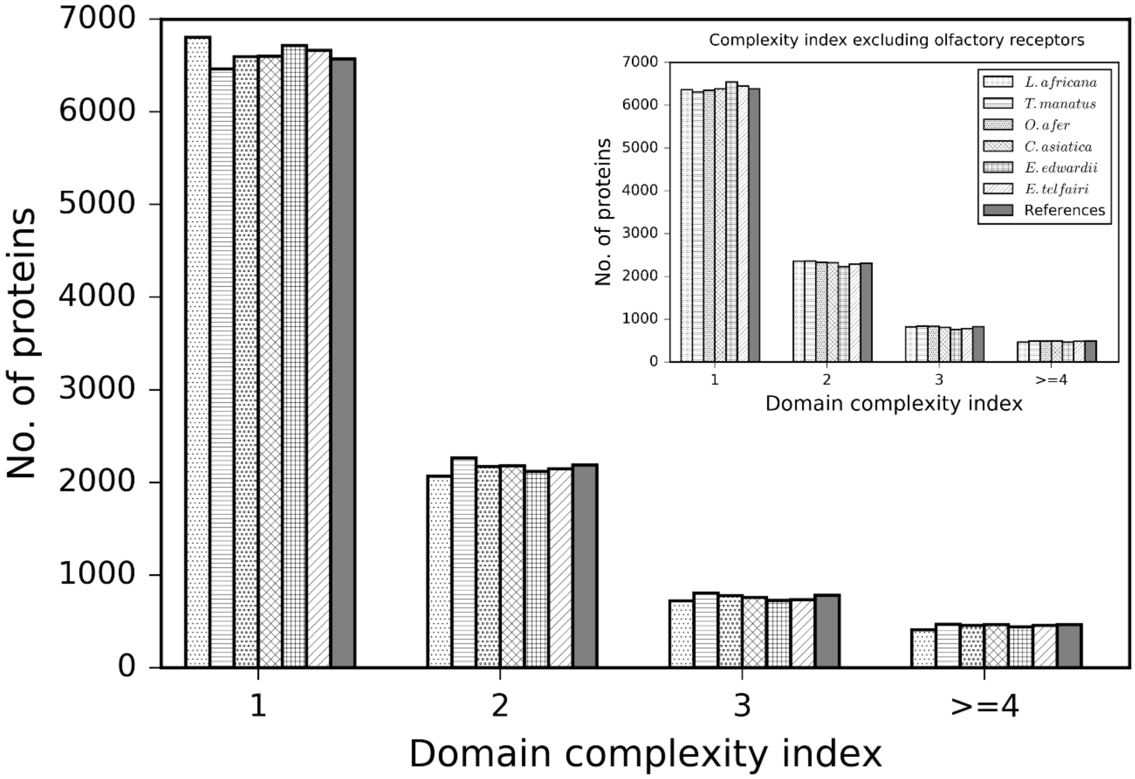

Supplementary figure S3. Structural domain associations in Afrotheria. (a) Abundance of structural domains based on SCOP 'class' observed in the Afrotheria. As in Figure S2, averaged data from reference proteomes (*H.sapiens*, *M.musculus*, *E.caballus* and *B.taurus*) are provided as a single 'Reference' category. Average proportion of each class in the total number of structural domains associated with the proteomes is given in the top panel. (b) Proportion of structural domains belonging to each structural class (except multi-domain class, see Figure 5a) that occur in single (grey) and multi-domain protein (gold) context.

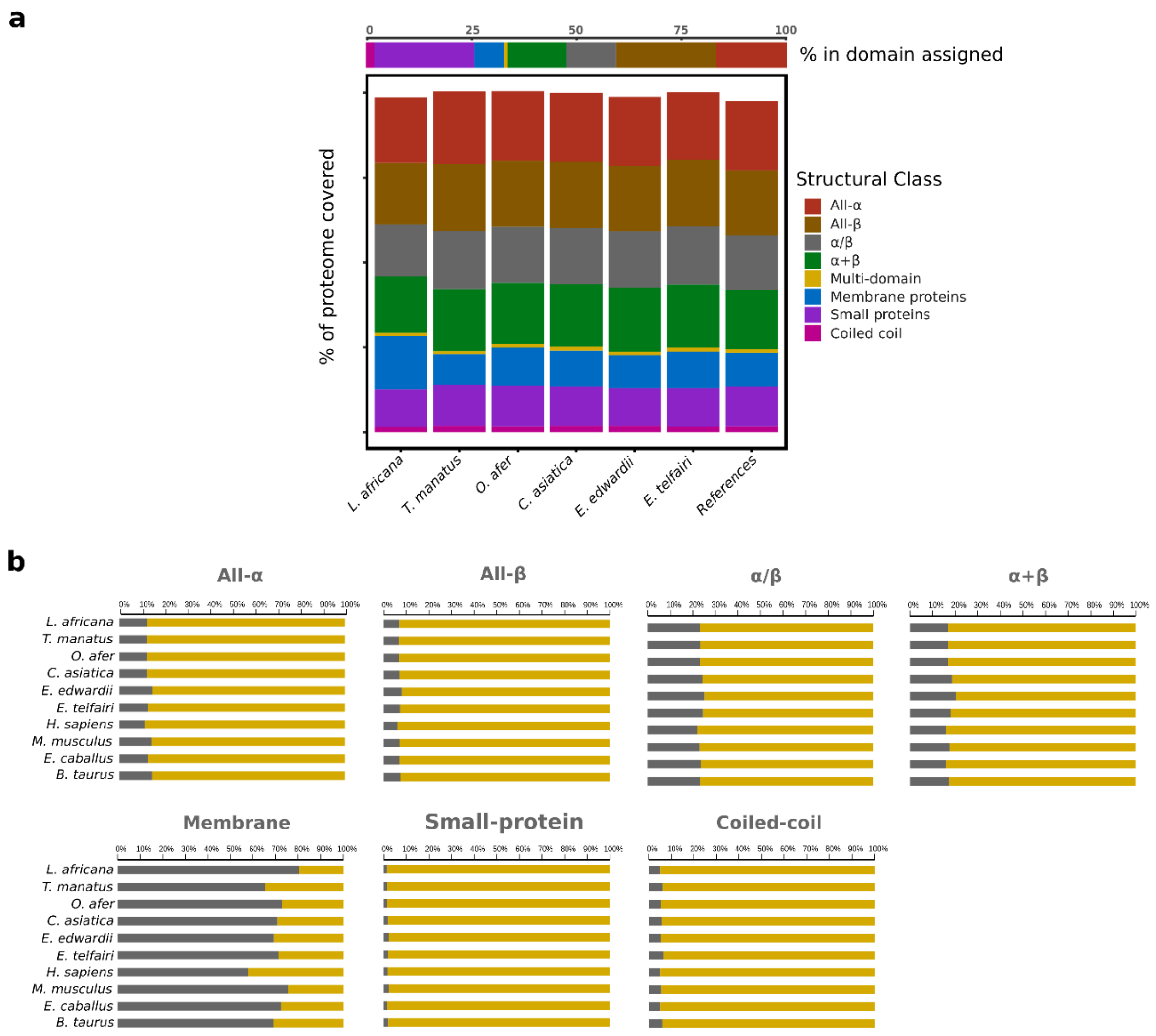

Supplementary Table legends

(Supplementary tables have been uploaded individually)

Supplementary Table S1. Statistics on sequence conservation of orthologous proteins in Afrotheria. Table S1A in Sheet 1 provides total number of most, moderate, and least conserved orthologous proteins in Afrotherian species. Table S1B-G in Sheets 2-7 is the list of Afrotherian proteins in each species and their orthologues.

Supplementary Table S2. Potential function of Afrotheria-specific proteins. Table S2A lists molecular features and inferred function of Afrotheria-specific uncharacterized proteins. Table S2B contains details on function prediction for species-unique proteins of Afrotheria.

Supplementary Table S3. Distribution of functional domains in Afrotheria and reference species. Table S3A lists the normalized frequency of distribution of various functional domains in each species. Tables S3B-D lists proteins showing domain complexity index of 2, 3 and 4 or more.

Supplementary Table S4. Functional domain architectures in Afrotheria. Table S4A lists unique functional domain architectures in Afrotheria. Table S4B provides the list of domain repeats showing unique domain combination in Afrotheria.

Supplementary Table S5. Structural domain distributions in Afrotheria. Table S5A provides the list of structural domain superfamilies and their numbers in various Afrotherian proteomes. Table S5B is a list of the most commonly occurring domains and number of their species- unique architectures in various Afrotherian proteomes. Table S5C lists species-unique architectures.

Supplementary Table S6. Domains that occur in high numbers in Afrotheria. Table S6A shows the distribution of MAGE domain proteins in Afrotheria. Table S6B shows the distribution of Ribosomal domains in Afrotheria and other daily torpor/hibernating species.
